# Supplementary material for: Genetic determinants of plasma protein levels in the Estonian population
Source: Sci Rep. 2024 Apr 2;14:7694. doi: 10.1038/s41598-024-57966-3 (PMC10987560; doi:10.1038/s41598-024-57966-3)
Supplement: Supplementary file 1 — Supplementary Figure S1. [file 41598_2024_57966_MOESM1_ESM.docx]

**Supplementary Figures**


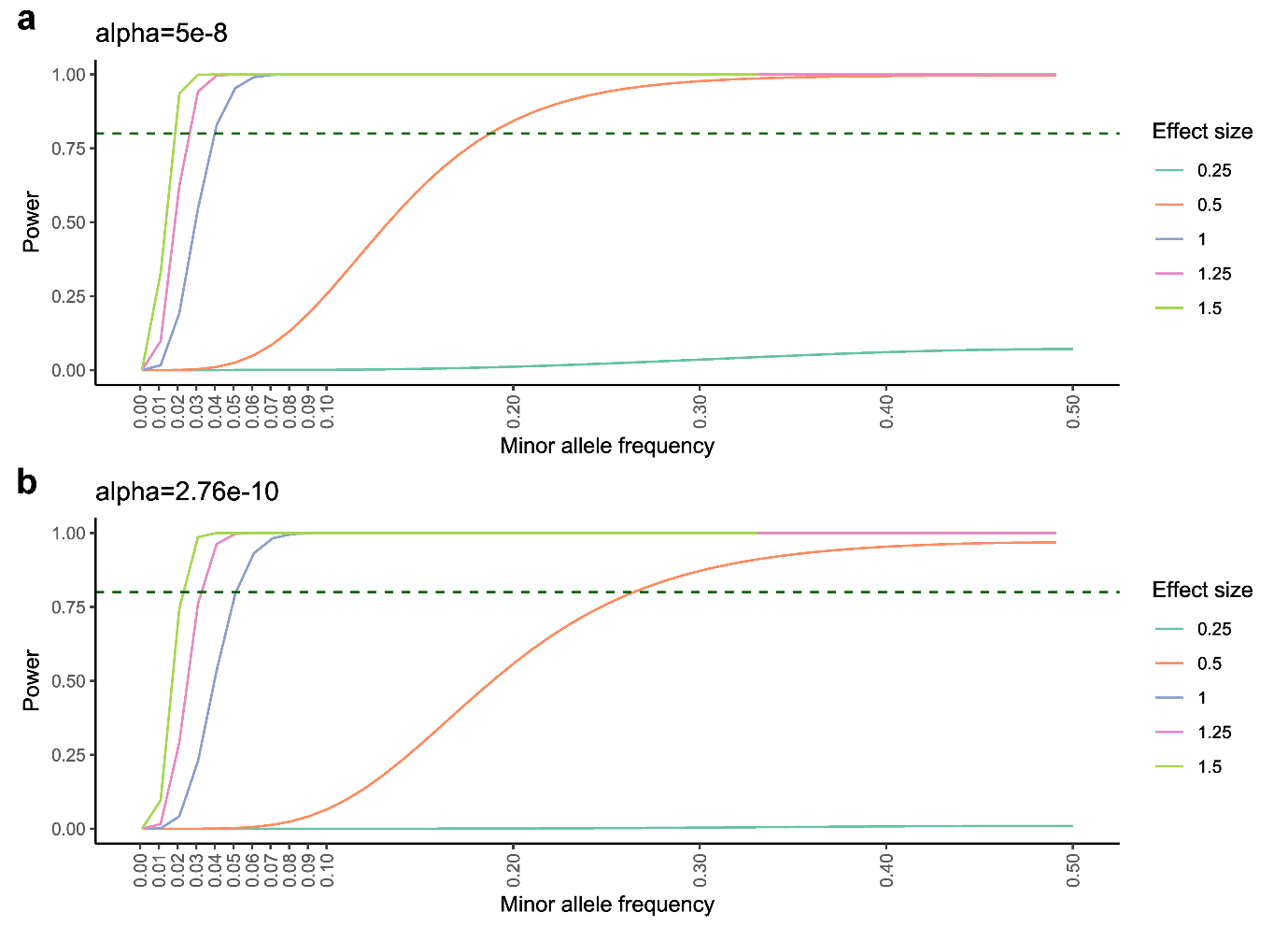


**Supplementary Figure S1. Power calculations in a cohort *n* = 500 for (a) α = 5 × 10^-8^ and (b) α = 2.76 × 10^-10^.** Each coloured continuous horizontal line represents power by minor allele frequency (MAF) and pQTL variant effect size. The green horizontal dashed line indicates a power of 0.8.
